# Treatment of Symptom Clusters in Schizophrenia, Bipolar Disorder and Major Depressive Disorder With the Dopamine D3/D2 Preferring Partial Agonist Cariprazine

**DOI:** 10.3389/fpsyt.2021.784370

**Published:** 2021-11-23

**Authors:** Borjanka Batinic, Ivan Ristic, Milica Zugic, David S. Baldwin

**Affiliations:** ^1^Clinic of Psychiatry, University Clinical Centre of Serbia, Belgrade, Serbia; ^2^Department of Psychology, Faculty of Philosophy, University of Belgrade, Belgrade, Serbia; ^3^Department of Epidemiology, Medical School, University of Belgrade, Belgrade, Serbia; ^4^Department of Psychiatry, Institute of Mental Health, Belgrade, Serbia; ^5^Clinical and Experimental Sciences (CNS and Psychiatry), Faculty of Medicine, University of Southampton, Southampton, United Kingdom; ^6^Department of Psychiatry and Mental Health, University of Cape Town, Cape Town, South Africa

**Keywords:** cariprazine, D3/D2 partial agonist, schizophrenia, bipolar I disorder, major depressive disorder

## Abstract

Cariprazine is currently approved for the treatment of patients with schizophrenia (USA and EU), and for manic, depressive, and episodes with mixed features in bipolar I disorder (USA): several randomized controlled studies have also explored its efficacy in patients with major depressive disorder. This review summarizes its current therapeutic uses and potential advantages for treating the main symptoms of schizophrenia, bipolar I and major depressive disorder, considering its pharmacodynamic properties, efficacy, and tolerability. Its predominantly D3 receptor preferring affinity, with functional selectivity according to the prevailing neuronal environment, contributes to its efficacy across a wide array of psychopathological symptoms (including reality distortion, disorganized thought, negative symptoms, mood disturbance, anhedonia, and cognitive impairment), and to a favorable side effect profile. Cariprazine may be a “drug of choice” in patients with predominant negative and cognitive symptoms of schizophrenia, as well as those with metabolic syndrome. Further investigation of its relative efficacy when compared to aripiprazole or other active comparators is warranted. Its effectiveness in the treatment of bipolar mania, bipolar I depression and bipolar I episodes with mixed features, with minimal accompanying metabolic changes is well-established. The longer half-life and delayed time to relapse in patients diagnosed with schizophrenia when compared to other second-generation antipsychotics represent other advantages, given the high rates of non-adherence and frequent relapses seen in clinical practice. Its efficacy in overlapping symptom domains in other major psychiatric disorders appears promising.

## Introduction

Schizophrenia, bipolar disorder, and major depression are common major mental disorders which make a substantial contribution toward total disability adjusted life years. Despite nosological specificity, these disorders can share mood disturbance (depression, euphoria, irritability, anhedonia, etc.), reality distortion (hallucinations, delusions), and cognitive impairment may be present even during periods of remission, exerting negative effects on social functioning, basic life skills and quality of life (1–3). This overlap differs according to the severity of individual symptoms and may be due to varying degrees of genetic contributions, as indicated by twin, family, and molecular genetic studies (4–8).

Due to the known actions of available psychotropic medicines on individual neuronal and biochemical mechanisms within the central nervous system, and to growing awareness of their effects across a wide range of overlapping symptoms, a current imperative for developing innovative treatments for patients with mental disorders is focused on compounds that combine multimodal activity with greater efficiency on different symptom profiles, potential neuroprotective effects, and modulatory effects on the course of the disorder.

With the advent of aripiprazole, a new class of antipsychotic drugs emerged that exhibit partial agonism at dopamine (DA) receptors, thus allowing adaptation to the prevailing transmitter environment, e.g., to act as either a functional antagonist or agonist (9–14). This modulation of dopaminergic transmission decreases DA levels when they are high or increases levels when they are low (9–14). In the case of schizophrenia, functional antagonism in mesolimbic pathways reduces positive symptoms of psychosis, whereas functional agonism in the nigrostriatal pathways reduces the possible development of iatrogenic extrapyramidal side effects (13, 14). Dopamine D3 receptors have a high affinity for DA and are localized predominantly in the ventral striatum and other parts of the limbic system, whereas their distribution is low in the dorsal (motor) striatum and cortical region (15). This distribution allows dopamine D3/D2 preferring partial agonist cariprazine to exert antipsychotic activity, with a low propensity for unwanted extrapyramidal side effects, hyperprolactinemia, metabolic syndrome, and anhedonia (12, 16).

## Cariprazine

Cariprazine is a “dopamine stabilizer”, with higher affinity toward D3 receptors than dopamine, thus increasing dopaminergic neurotransmission in the nucleus accumbens and hippocampus. It binds to D3 receptors with a 10-fold higher affinity than for D2 receptors, exerts antagonist effects at 5-HT_2B_ receptors and partial agonism at 5-HT_1A_ receptors, and has moderate and low activity for histamine H1 and 5-HT_2C_ receptors, respectively (12). Cariprazine was granted approval from the Food and Drug Administration (FDA) in the United States for the treatment of schizophrenia in adults as well as for patients with bipolar I disorder experiencing symptoms of acute mania, mania with mixed features, or depression (17). Furthermore, randomized controlled trials (RCTs) are currently exploring its efficacy as add-on therapy in patients with major depressive disorder (18).

### Metabolism and Interactions

Cariprazine is metabolized via P450 3A4 (CYP3A4) and to a lesser extent by cytochrome P450 2D6 (CYP2D6) into two major active metabolites, desmethyl-cariprazine (DCAR) and didesmethyl-cariprazine (DDCAR) with broadly similar pharmacological activity (19). Time to reach steady state based on half-life is 2–4 days for cariprazine, and 1–2 days and 1–3 weeks for DCAR and DDCAR, respectively (20). This contrasts with the half-life parameters of other oral second-generation antipsychotics, with shorter half-lives between 3 and 91 h (e.g., risperidone, paliperidone, aripiprazole, asenapine, brexpiprazole, iloperidone, lurasidone, olanzapine, paliperidone, quetiapine, and ziprasidone) (21, 22).

In patients undergoing treatment with concomitant carbamazepine and oxcarbazepine, the effect of cariprazine will be decreased due to carbamazepine and oxcarbazepine induction of cytochrome P450 CYP3A4 enzyme, and this combination is contraindicated, as well as combination with other P450 CYP3A4 inducers (e.g., phenobarbital, phenytoin, rifampicin, St. John's Wort, and glucocorticoids) (23). When a patient is taking strong inhibitors of P450 CYP3A4 **(**e.g., clarithromycin, erythromycin, diltiazem, itraconazole, ketoconazole, ritonavir, verapamil, goldenseal, and grapefruit**)** the dose of cariprazine should be reduced by half. No dosage adjustment is necessary if patient is concomitantly taking CYP2D6 inhibitors (23).

## Cariprazine Efficacy Across the Wide Range of Symptoms of Schizophrenia

The principal clinical features of schizophrenia comprise positive and negative symptoms, mood symptoms, disorganization symptoms and cognitive impairments, with different underlying pathophysiological mechanisms and different responses to pharmacological treatments (24, 25). First- and second-generation antipsychotics can both successfully attenuate the positive symptoms of schizophrenia, however the negative symptom domain (dominated by the “5As”- apathy, amotivation, anhedonia, alogia, asociality) still represents an unmet clinical need as many currently available antipsychotics fail to improve these (26).

Cariprazine has shown efficacy in reducing the total Positive and Negative Syndrome Scale (PANSS) as well the Clinical Global Impression-Severity (CGI-S) scale scores in several double-blind, placebo-controlled clinical trials involving patients with acute exacerbation of schizophrenia (27–29). No clinically significant changes compared to placebo were observed regarding metabolic parameters, ECG abnormalities (including QT prolongation), laboratory results or prolactin levels (27–29). The most common side effects throughout the studies were akathisia, extrapyramidal disorder, tremor, insomnia, sedation, dizziness, and gastrointestinal side effects (27–29). The pooled-analysis reported by Marder et al. (30) which investigated data from three positive, 6-week duration, double-blind, placebo-controlled, phase 2/3 trials of cariprazine in patients with acute exacerbation of schizophrenia has shown its efficacy in doses of 1.5, 3, 4.5 and 6 mg in treating a wide array of symptoms (positive symptoms, negative symptoms, disorganized thought, uncontrolled hostility/excitement, and anxiety/depression). Furthermore, when compared to placebo, cariprazine in daily doses of 1.5, 3 and 6 mg a day has shown efficacy in relapse prevention (31), as well as in doses of 3, 6 and 9 mg (32). Although clinical studies have examined doses above 6 mg per day, the manufacturer's recommendation is a maximum of 6 mg per day due to the dose-dependent occurrence of adverse events and insufficiently increased efficacy to justify higher dosage (23).

Cariprazine was also found to be uniquely effective in treating primary negative symptoms of schizophrenia, due to its predominant preference for D3 receptors, as well as its considerable affinity for 5-HT_1A_ receptors (33–37). In a randomized, double-blind multicenter study which compared the efficacy of cariprazine and risperidone in patients with schizophrenia with predominantly negative symptoms, cariprazine in the mean dose of 4.2 mg/d showed a superiority over risperidone (mean dose 3.8 mg/d) at Week 14, which was continued until Week 26 (end of trial) (33). Significant improvement over risperidone was evident on a wide array of negative symptoms of schizophrenia (35). In therapeutic doses of 4.5 mg−6 mg/d, cariprazine has shown greater efficacy when compared to aripiprazole in reducing moderate to severe negative symptoms of schizophrenia (36). An observational study by Rancans et al. (34) showed the effectiveness of cariprazine in ‘real-life' clinical settings when treating negative symptoms. In a *post-hoc* analysis of long-term treatment, studies have shown that cariprazine also improved everyday functioning and social skills which influence quality of life (37). This is a potentially significant advantage, considering that recovery should include not only symptom reduction but also functional improvement across various aspects of life (38, 39).

### One Day Dosing of Cariprazine

Due to the long half-life of cariprazine once daily administration is possible, which facilitates dosing and treatment adherence (40). The long half-life of cariprazine and its active metabolites, especially (DDCAR) with its 1–3-week half-life (20) distinguish cariprazine from other second-generation antipsychotics whose half-life, including their metabolites, is <4 days. It may be an important factor in the delayed relapse rate when compared to other second-generation antipsychotics (placebo relapse rate after last dose of cariprazine at week four was estimated to be 5%, compared to other oral antipsychotics ranging from 8–34% (22). Treatment with cariprazine can thus bridge the problem of emerging non-adherence after hospital discharge and first outpatients' appointments, as non-adherence and partial adherence is still a substantial problem which threaten patients' recovery (41).

### Cariprazine as an Add-on Therapy to Other Antipsychotics in Patients With Schizophrenia, and Switching Recommendations

Early and late non-response is common in schizophrenia, and several potential management strategies are proposed, among them the adding-on of another medication. Two case reports by de Berardis et al. (42) have shown that adding cariprazine to 400 and 300 mg of clozapine (in an initial dose of 1.5 mg and after 7 days titration 3 mg a day) resulted in an improvement in total PANSS score, positive and negative sub-scores, and general symptoms score: the combination was well-tolerated, with no side effects, and reduction in some of the weight gained during clozapine therapy.

According to the recommendation from an International Panel reported by Fagiolini et al. (43) when switching from other antipsychotic drugs to cariprazine, a cross-titration is recommended with the need to reach an effective dose (“plateau” in plasma concentration) of cariprazine before tapering or stopping the first medication; furthermore, adding benzodiazepines (e.g., lorazepam) for a short period may also help in reducing rebound of symptoms; the length of the titration depends on the type of other antipsychotic, which in the case of aripiprazole is one week or less, 2–3 weeks if the main antipsychotic was risperidone or haloperidol, and longer (3–4 weeks) in the cases of olanzapine and quetiapine.

## Effects of Cariprazine on Neurocognitive Deficits in Patients With Severe Mental Disorders

Patients with severe mental disorders such as schizophrenia, bipolar disorder and major depression often have impairments across many cognitive domains, such as memory, motor speed, verbal fluency, executive function, attention, speed of information processing, and affective memory (3, 44–47), These neurocognitive impairments can limit work performance and social adjustment, reduce overall quality of life as well as are the best predictors for long-term psychosocial outcomes (2, 48). Given that neurocognitive deficits are often also present during periods of remission (3, 49, 50) the importance of addressing neurocognitive function is clear, especially in the early stages of the illness. While DA antagonists regulate DA excess and attenuate positive psychotic symptoms, they may also increase negative symptoms, cognitive problems, and extrapyramidal side effects by blocking activity in the regions characterized by dopaminergic underactivity (51, 52). Animal studies have demonstrated the effects of cariprazine as D3 preferring D3/D2 partial agonists in striatum, nucleus accumbens and ventral hippocampus, parts of the brain important in linking memory of the surrounding and motor behavior when recalling information about reward-seeking behavior (16). Furthermore, cariprazine significantly diminishes phencyclidine (PCP)-induced cognitive deficits in *in vivo* animal studies, via a D3 receptor activity (53, 54). Importantly, it is suggested that compounds which have partial agonism at D3 receptors function as functional agonists in brain regions with a relative deficit in dopamine, hence causing pro-cognitive effects (53, 55). Therefore, drugs with such mechanism of action can be an important driver for functional recovery in patients with schizophrenia, bipolar I disorder or even major depressive disorder (55–58).

## Cariprazine Efficacy Across the Wide Range of Symptoms of Bipolar Disorder

Bipolar I disorder is a chronic mental illness presenting with episodes of manic, depressive mood and episodes with mixed features (24), with a significantly negative impact on main domains of quality of life (59), which is lower compared to healthy controls even in the period of euthymia (60). The Diagnostic and Statistical Manual of Mental Disorders-5th edition (24) specifies episodes with mixed features in both Bipolar I and Bipolar II disorder, in which at least three symptoms of opposite polarity are present at the same time with major mood disturbance. This specifier promises recognition and treatment of patients with mixed features, characterized with an unfavorable course with more severe symptomatology, more frequent mood swings, higher rates of comorbidity (61), and suicidal behavior (62).

Double-blind, placebo-controlled studies have demonstrated the efficacy of cariprazine in low doses (3–6 mg/day) and high doses (6–12 mg/day) in the treatment of manic and mixed episodes of bipolar I disorder (63–66). Importantly, cariprazine was well-tolerated with non-significant changes in prolactin levels, QT intervals and metabolic parameters [with the exception for fasting glucose in the study by Durgam et al. (64)]. Akathisia and other extrapyramidal symptoms were the only prominent side effects when compared to placebo (63–65). In a *post-hoc* pooled analysis of three studies by McIntyre et al. (66) cariprazine was efficacious in significantly reducing manic and depressive symptoms in Bipolar I mania with mixed features. The dose range recommended in the treatment of acute manic or acute mixed episodes is 3–6 mg a day, with a starting dose of 1.5 mg a day and increase to 3 mg on Day 2 (23).

In bipolar I disorder patients are spend much of their time in depressive states (67). Antidepressant monotherapy in the treatment of bipolar depression is not recommended as it can induce mania or hypomania as well as increase mood cycling (68). Double-blind, placebo-controlled studies have shown efficacy of cariprazine in reducing depressive symptoms in bipolar I depression (69, 70). Cariprazine in a dosage of 1.5 mg/d had the most robust efficacy and good safety for the treatment of patients with Bipolar I depression (69). Maximum recommended dose is 3 mg a day, increased on Day 15 (23). In a *post-hoc* analysis of pooled data from three randomized, double-blind, placebo-controlled studies, cariprazine was found to be effective across an array of symptoms of bipolar I depression such as sadness, fatigue, and anhedonia (expect inner tension) in the doses of 1.5 and 3 mg in adult patients (71). In addition, cariprazine might be also effective in improving anhedonia and cognitive dysfunction (72). In terms of safety, findings from a pooled analysis of four studies in patients with bipolar depression (73) showed that cariprazine in doses of 1.5 and 3.0 mg is well-tolerated with little weight gain and minimal metabolic changes when compared to placebo. The most common side effects experienced by patients were akathisia, restlessness, nausea, and fatigue. Importantly, cariprazine did not destabilize mood or induce manic switch. Furthermore, incidence of suicidality was low in cariprazine group (73). Finally, a *post-hoc* analysis of three randomized, placebo-controlled studies showed that cariprazine is effective in treating bipolar I depression with mixed features in the doses of 1.5 and 3.0 mg per day (74).

Particular challenges when treating patients with bipolar disorder occur in patients with rapid cycling conditions, background dysregulated affective temperaments, a history of suicidality, as well as elderly patients (75–78). Another problem resides in the frequent need for polypharmacy (79). Cariprazine can be combined with the mood stabilizer lithium carbonate but combination with carbamazepine is contraindicated, as carbamazepine is a strong inducer of cytochrome P450 CYP3A4 (23). Further research can answer whether cariprazine treatment is beneficial in addressing these pressing challenges.

Other than quetiapine, cariprazine is the only drug to receive FDA approval for both acute treatment of mania and acute depressive episodes associated with Bipolar I disorder. The dosage approved for use in bipolar depression ranges from 1.5–3 mg/day. However, the European Medicines Agency (EMA) has not currently approved cariprazine for this indication.

## Efficacy of Cariprazine as an Augmentation Approach in Major Depressive Disorder

Despite the availability of multiple classes of antidepressants, treatment of patients with major depression is often a challenge, and a significant proportion of patients have an inadequate acute and long-term response to antidepressant treatment, with much “room for improvement” (80). The sub-optimal efficacy of current antidepressant treatment and high rates of treatment resistant cases underlie the need for frequent alternative solutions, such as adding a second-generation antipsychotic with antidepressant effects.

Although not approved by the FDA for the treatment of major depression, some double-blind placebo-controlled studies have indicated the efficacy of cariprazine as an adjunctive therapy to antidepressants in treatment-resistant major depressive disorder (81, 82). By contrast, the randomized-controlled, double-blind study reported by Earley et al. (83) did not find significant improvement with adjunct cariprazine in patients diagnosed with major depressive disorder with previously inadequate response to antidepressant treatment. The meta-analysis undertaken by Vázquez et al. (84) found that cariprazine is more effective than placebo, but less effective than aripiprazole [olanzapine + fluoxetine] combination, risperidone, and ziprasidone in attaining additional antidepressant response in patients diagnosed with major depressive disorder with inadequate response: akathisia was the most frequent side effect. When combining cariprazine with SSRIs or SNRIs clinicians should be aware of side effects such as akathisia, insomnia, and nausea, especially in doses of cariprazine higher than 2 mg/d (81). Further research is needed to fully clarify the role of cariprazine as an augmentation in the treatment of patients with unipolar depression who previously failed to respond to antidepressants.

## Discussion

Seventy-two years have passed since the appearance of haloperidol as the main representative of the “first generation” or “typical” group of antipsychotics characterized by activity through antagonism of dopamine D2 receptors. Some progress has been made with “second generation” or “atypical” drugs which achieved their effectiveness through potent binding to 5-HT_2A_ receptors as well as to D2 receptors. Current “dopamine modulators” (e.g., aripiprazole, brexpiprazole, blonaserin) differ depending on their predominant site of action at dopamine and serotonin receptors, with consequent differences in the spectrum of psychopathology they reduce and their side effect profile (55). Among them, cariprazine has a D3 receptor preferring affinity, with functional selectivity according to the prevailing neuronal environment, thus contributing to its efficacy across an array of symptoms of schizophrenia, bipolar disorder, and major depressive disorder (27–37, 63–66). Importantly, cariprazine also exhibits a favorable side effect profile with no higher incidence on metabolic parameters, ECG abnormalities, vital sign and prolactin levels changes compared to placebo (27–37, 63, 65, 66). Its efficacy in long term use in schizophrenia is also confirmed (31, 32, 37).

Cariprazine may be a “drug of choice” in patients with predominant negative and cognitive symptoms of schizophrenia, as well those with metabolic syndrome. Its effects as an add-on approach to potentially reverse side effects caused by other medications should be further investigated. Due to the long half-life of cariprazine and its active metabolites, the time to relapse in patients diagnosed with schizophrenia is delayed when compared to other neuroleptics, which represents a useful advantage in clinical practice. Its effectiveness in treatment of bipolar mania, bipolar depression, and bipolar episodes with mixed features with minimal metabolic changes is well-established, and core symptoms of bipolar depression -sadness, fatigue, and anhedonia, as well as cognitive deficit have been strongly reduced by cariprazine, without switching to mania.

Given the efficacy of cariprazine in the treatment of core behavioral, mood and cognitive symptoms of severe mental disorders, it is reasonable to anticipate potential benefits in the treatment of at least some symptoms in autism spectrum disorder and addictions. Preliminary pre-clinical and clinical studies have shown beneficial effects of cariprazine in reversing core behavioral deficits and behavioral disturbances—aggression, irritability, self-injurious behavior, and impulsivity—in autism-spectrum disorder, as well as in “relapse prevention” in cocaine-seeking rats (85–87).

## Conclusions

The dopamine D3/D2 preferring partial agonist cariprazine has shown efficacy across the wide array of symptoms in schizophrenia and bipolar disorder (reality distortion, disorganized thought, mood disturbance, anhedonia, and cognitive impairment), and a favorable side effect profile. Its efficacy in overlapping symptom domains in patients with other major psychiatric disorders (major depression, autism spectrum disorder, addictions, etc.) is worthy of further exploration.

## Author Contributions

BB wrote the first and subsequent drafts of the manuscript. IR and MZ assisted in literature research and writing the manuscript. DB supervised and revised all later drafts of the paper. All authors reviewed the paper and approved the final version.

## Conflict of Interest

The authors declare that the research was conducted in the absence of any commercial or financial relationships that could be construed as a potential conflict of interest.

## Publisher's Note

All claims expressed in this article are solely those of the authors and do not necessarily represent those of their affiliated organizations, or those of the publisher, the editors and the reviewers. Any product that may be evaluated in this article, or claim that may be made by its manufacturer, is not guaranteed or endorsed by the publisher.
